# M. Thomas on Colica Pictonum

**Published:** 1826-07

**Authors:** 


29. *Colica Pictonum.* A French writer (M. Thomas de trois vevres) has lately published some observations on the seat of this curious disease. It is unquestionable that the sensorium very often presents disturbance of function in this complaint; but then it has usually been considered as symptomatic of the abdominal affection. M. Thomas is of a different opinion, and thinks that, in the majority of cases, the disease commences with cerebral symptoms. In the dissection of eleven cases, he found injection of the meninges, softenings and other morbid appearances in the cerebral structure, with serous or sanguineous extravasations between the membranes. In 275 individuals affected by *metallic emanations*, 92 had not the abdominal affection. This we are ready to grant; but if the disease be confined to the emanations of *lead*, we believe that a small proportion will be deficient of the common enteric phenomena. No doubt the vapours of all metallic substances must act more or less on the brain and nervous system generally, and we need hardly wonder that such appearances as are described by M. Thomas, should be found after death in the cranial cavity. But these circumstances do not, we apprehend, amount to any just reason why we should alter the pathology of colica pictonum, or unhinge our ideas respecting the treatment of the disease.—*Jour. Univers.*

